# Molecular Targets of Plant-Derived Bioactive Compounds in Oral Squamous Cell Carcinoma

**DOI:** 10.3390/cancers16213612

**Published:** 2024-10-26

**Authors:** Gabriela Mitea, Verginica Schröder, Irina Mihaela Iancu, Horațiu Mireșan, Valeriu Iancu, Laura Adriana Bucur, Florin Ciprian Badea

**Affiliations:** 1 Department of Pharmacology, Faculty of Pharmacy, Ovidius University of Constanta, 900470 Constanta, Romania; gabriela.mitea@365.univ-ovidius.ro; 2 Department of Cellular and Molecular Biology, Faculty of Pharmacy, Ovidius University of Constanta, 900470 Constanta, Romania; 3 Department of Toxicology, Faculty of Pharmacy, Ovidius University of Constanta, 900470 Constanta, Romania; horatiu.miresan@univ-ovidius.ro; 4 Department of Pharmaceutical Technology, Faculty of Pharmacy, Ovidius University of Constanta, 900470 Constanta, Romania; valeriu.iancu@univ-ovidius.ro; 5 Department of Pharmacognosy, Faculty of Pharmacy, Ovidius University of Constanta, 900470 Constanta, Romania; laurabucur@univ-ovidius.ro; 6 Department of Dental Medicine, Faculty of Dental Medicine, Ovidius University of Constanta, 900684 Constanta, Romania; florin.badea@365.univ-ovidius.ro

**Keywords:** cancer, oral squamous cell carcinoma, phytochemicals, molecular processes, complementary therapies, phytotherapy

## Abstract

Background: With a significant increase in both incidence and mortality, oral cancer—particularly oral squamous cell carcinoma (OSCC)—is one of the main causes of death in developing countries. Even though there is evidence of advances in surgery, chemotherapy, and radiotherapy, the overall survival rate for patients with OSCC has improved, but by a small percentage. This may be due, on the one hand, to the fact that the disease is diagnosed when it is at a too-advanced stage, when metastases are already present. Methods: This review explores the therapeutic potential of natural herbal products and their use as adjuvant therapies in the treatment of oral cancer from online sources in databases (PubMed, Web of Science, Google Scholar, Research Gate, Scopus, Elsevier). Results: Even if classic therapies are known to be effective, they often produce many serious side effects and can create resistance. Certain natural plant compounds may offer a complementary approach by inducing apoptosis, suppressing tumor growth, and improving chemotherapy effectiveness. The integration of these compounds with conventional treatments to obtain remarkable synergistic effects represents a major point of interest to many authors. This review highlights the study of molecular mechanisms and their efficiency in in vitro and in vivo models, as well as the strategic ways in which drugs can be administered to optimize their use in real contexts. Conclusions: This review may have a significant impact on the oncology community, creating new inspirations for the development of more effective, safer cancer therapies with less toxic potential.

## Simple Summary

Oral cancer represents a main public health issue around the world due to the mortality rates associated with it, the lack of effective and potent drugs, the multitude of side effects and the expensive treatments. Until now, no natural alternative therapeutic methods have been found beneficial enough to replace conventional drugs. Thus, bioactive compounds with anticancer properties can represent a necessary alternative through a targeted molecular approach to the multiple pathways involved in carcinogenesis. This review explores how the therapeutic potential of natural compounds can be harnessed and how they can be used as adjuvant therapies for oral cancer treatment. The collected results focused only on plant extracts and biologically active metabolites known for their antioxidant, anti-inflammatory and antitumor potential, which can be described as future chemopreventive agents against oral cancer, alone or in therapeutic combinations.

## 1. Introduction

Oral cancer (OC), included in the category of head and neck malignancies, is one of the ten most common cancers in the world, with a 5-year survival rate for patients diagnosed with this type of cancer [1,2,3,4]. It remains a worldwide concern for the preservation of human health due to late diagnosis, low cure rate and high mortality [1,2,5]. Although oral cancer may not be the most aggressive in terms of metastatic potential, it carries a high mortality rate due to late diagnosis and recurrence [6,7]. Risk factors for oral cancer include alcohol and tobacco use, chronic inflammation, human papillomavirus (HPV) infection, fungal infection and chronic periodontitis, ultraviolet radiation, immunosuppression and diet [8,9,10,11] with varying contributions based on cancer subtype. In addition to these factors, the variety of gene alterations that occur moderately over time, together with the ability of the tumor to evade the host’s immune system, is also an important risk factor [12].

Many published studies have evaluated cell cycle markers, immune response, apoptosis and angiogenesis, as well as adhesion and matrix-degradation-related molecules [13]. Oncogenic signaling pathways, including the EGFR pathway, PI3K/AKT/mTOR pathway, Wnt/β-catenin pathway, JAK/STAT pathway, MET pathway and RAS/RAF/MAPK pathway, are aberrantly activated and upregulated and involved in OSCC progression [14].

Chemotherapy, radiotherapy, surgery [1], immunotherapy and hormonal therapy are the most common treatment methods used to treat oral cancer [15]. To ameliorate pathologic conditions, many oral cancer patients are successfully treated with adjuvant therapies or anticancer drugs, but these treatments are highly toxic to normal cells and tissues and can lead to the onset of many adverse effects [16]. Most side effects and complications differ depending on the oncologic treatment chosen by the specialist. Surgical treatment can have a negative impact on speech, swallowing, mastication processes and can culminate in cranial nerve damage, neurological problems and issues related to facial aesthetics [17]. Acute complications associated with chemotherapy and radiation therapy include dysphagia, dysgeusia, xerostomia, candidiasis, and mucositis, while chronic complications can generate trismus, radiation caries, and osteonecrosis [18]. Therefore, there is an urgent need for an innovative therapeutic approach that can improve treatment outcomes and life quality for patients [12,19]. One of the promising alternative complementary therapies for the treatment and management of human cancers is based on active phytocompounds. The common mechanism by which anticancer drugs extracted from medicinal plants can exert their cytotoxic effects seems to invariably induce cell cycle arrest and cause cell death through the process of apoptosis [20].

Recent advances in discovering and understanding the mode of action for these phytocompounds have revealed a wealth of opportunities that could revolutionize medicine, nutrition and even the environment [21]. Bio-active phytoconstituents are generally harmless, with a minimal number of adverse reactions, being more effective due to high absorption and rapid metabolization in the body. Another advantage is the increased patient compliance based on traditional and cultural knowledge. The accessibility of natural resources allows for reduced treatment costs by facilitating purification and standardization processes for use in modern drug delivery systems [22]. Some of the important phytochemicals are carotenoids, polyphenols, phytosterols, saponins, isoprenoids and certain polysaccharides. These phytochemicals exhibit strong antioxidant, antimicrobial and antiviral properties. They also help regulate gene transcription, improve gap junction communication and enhance the body’s immunity [23].

Curcumin, a polyphenol present in turmeric rhizome, shows potential activity in the treatment of cancer through pleiotropic mechanisms of action that simultaneously influence several pathways [24]. The antineoplastic effects of resveratrol, a polyphenolic antioxidant, as well as quercetin, a flavonol, on different types of cancer have been reported in various in vitro studies. These effects were obtained by significantly reducing cell viability and inducing autophagic cell death in oral cancer cells, but not in normal cells. In addition, quercetin also possesses an anti-metastatic effect by regulating the expression of epithelial–mesenchymal transition (EMT)-related gene expression in oral cancer cells [25,26]. Although these products have shown promising properties for alternative therapies, their therapeutic efficacy is limited by their poor absorption and bioavailability.

Cancer chemoprevention using natural phytochemicals involves an emerging strategy to prevent, suppress, delay or cure cancer [27]. Chemoprevention of oral cancer will be directed towards its goal by understanding the pathogenesis of oral cancer, more effective methods to identify high-risk populations, monitoring of appropriate biomarkers and the discovery of novel chemopreventive agents [28].

This review covered the latest research in cancer chemoprevention and treatment using bioactive compounds from natural plants. The relevant molecular mechanisms involved in the pharmacologic effects of these compounds are discussed, which were based on the existence of complex molecular mechanisms, such as the NF-κB, MAPK and PI3K/AKT pathways, which were the basis of the inclusion criteria for this review. Recent developments of bioactive compounds for oral cancer management and their involvement in apoptosis induction, cell proliferation, oxidative stress response and key signaling pathways are also discussed. This review also focused on the synergistic beneficial effect of natural compounds and conventional chemotherapeutic agents. This would lead to the development of new leads and a futuristic approach that would prompt new and effective studies that could contribute to the development of new therapeutic strategies against OC.

## 2. Materials and Methods

The method involved the collection of all conclusive information from multiple online sources in several databases (PubMed, Web of Science, Google Scholar, Research Gate, Scopus, Elsevier) to identify from relevant studies the importance of the use of medicinal plants and natural compounds in the treatment of oral cancer, using keywords such as “oral cancer”, “phytochemicals” and “phytotherapy”. The collected studies highlight all types of plants and natural compounds with anticancer and chemoprotective properties used in oral cancer treatment. The chosen studies also focused on molecular mechanisms, apoptosis induction, proliferation inhibition and metastasis-related processes.

## 3. Types and Determining Factors of Oral Cancer

Cancer remains the second leading cause of death worldwide after heart disease, accounting for an estimated 9.6 million deaths [29,30]. The current situation is extremely worrying, with studies showing that one in four people worldwide is at risk of developing cancer during their lifetime [31]. Cancer is one of the fastest-growing diseases of the 21st century [31], with high incidence in both developed and developing countries [32]. Population registry data from the National Population-Based Cancer Registry Program indicate that the main cancer sites are the oral cavity, lungs, esophagus, and stomach in men and the cervix, breast, and oral cavity in women [33]. Oral cancer (OC), which refers to malignant neoplasms that originate in the oral cavity, is also a significant global health concern [34,35]. Oral squamous cell carcinoma (OSCC), the most common malignant neoplasm of the oral cavity, is the sixth most common cancer worldwide [3,31]. Cancer of the oral cavity should be particularly differentiated from oro-pharyngeal malignancy, in which the etiology, clinical presentation and treatment modalities vary considerably [36]. Oral cancer appears as a small, unfamiliar and unexplained growth or sore in the parts of the mouth including the lips, cheeks, tongue, sinuses, hard and soft palate, base of the mouth extending to the oropharynx [37].

Many primary cancers of the oral cavity are usually asymptomatic, whereas advanced lesions may present as ulcerations, with prominent, rigid margins, lasting more than three weeks. Ulcerations may be associated with erythroplakia (red spot), leukoplakia (white spot), or leukoplakia with mixed (red/white) spot. Pain is often absent until the late stages of the disease [37,38,39]. Symptoms may include pain or difficulty chewing or swallowing, hoarseness of voice [7].

The buccal cavity is a part of the upper aerodigestive tract that begins at the lips and ends at the anterior surface of the faucial arch [33]. These different areas of the oral cavity are lined with stratified squamous epithelial cells that are susceptible to the development of cancer [16]. The oral epithelium contains keratinized areas composed of stratified keratinized and non-keratinized squamous epithelium. The oral cavity and pharynxes are covered with non-keratinized stratified epithelium in areas less exposed to wear, such as the mucosa of the cheeks and lips. In areas of the oral cavity that are exposed to greater wear, such as the hard palate and gums, the stratified squamous epithelium is keratinized [16,40,41,42,43].

### 3.1. Types of Oral Cancer and Symptoms

Approximately 90% of oral cancers are oral squamous cell carcinomas [44], while 10% are malignant melanomas, soft tissue sarcomas, salivary gland tumors, non-Hodgkin’s lymphomas and metastases from other primary tumors (Table 1) [45]. Adenoid cystic carcinoma, mucoepidermoid carcinoma and low-grade polymorphic adenocarcinoma are types of minor salivary gland carcinomas that develop in the glands lining the mouth [45].

#### 3.1.1. Oral Melanoma

Oral melanoma is a metastatic, aggressive, lethal cancer with a very low incidence (0.2–8%) among all malignant melanomas [8]. Melanoma is a type of cancerous tumor resulting from uncontrolled proliferation of pigment-producing cells called melanocytes. This type of cancer commonly affects males and occurs mostly in the mucous membrane of the jaw, frequently on the keratinized mucous membrane of the hard palate and gums [8,46]. Risk factors associated with the development of oral melanoma are smoking, daily alcohol consumption, HPV (human papillomavirus) infection, sun exposure, comorbidities, transplantation, patients with other skin malignancies, and genetic factors [46,47]. The clinical appearance of oral melanoma is varied. However, it usually presents as a black-brown spot, macule or nodular lesion with varying shades of gray, red, purple or areas of depigmentation [48], and pain may be the most recent manifestation of melanoma [46]. The most common cell signaling pathway in melanoma progression by regulating cell proliferation, differentiation and survival is the RAS-RAF-MEK-ERK pathway, also known as the MAPK/ERK pathway. This signaling cascade involves the BRAF gene [49].

#### 3.1.2. Lymphoma

Lymphoma is a heterogeneous malignant disease of the lymphatic system resulting from a proliferation of lymphoid cells or their precursors [8,45,50]. Primary sites of oral lymphoma manifestations in the oral cavity include the lips, cheek, tongue, gums, buccal floor, palate [8]. Lymphomas are the third most common type of cancer occurring in the oral cavity and constitute 3% of malignant tumors [50]. Depending on histological changes, behavioral changes and aggressiveness, lymphomas can be divided into Hodgkin lymphoma (HL) and non-Hodgkin lymphoma (NHL), the latter group comprising B-cell, T-cell and NK-cell lymphomas [8,51]. The main clinical manifestations reported in oral lymphoma are swelling, pain, erosions, ulcers and numbness [50,52]. Oral manifestations specific for HL are rare and those for NHL may present as nodal or extranodal lymphoma [51].

Risk factors for NHL are immunosuppression (especially in AIDS patients), autoimmune and chronic inflammatory disorders, ultraviolet radiation, viruses and other pathogens (hepatitis C virus, HPV-human papillomavirus, *Helicobacter pylori*) (Table 1), exposure to pesticides such as organophosphate and organochlorine compounds [50,53]. Genetic factors often predominate; both double and triple translocation are involved in the progression of lymphoma. Double translocation refers to the translocation of C-MYC with other concomitant breakpoints, such as BCL2, BCL6, BCL1 or BCL3. Triple translocation consists of C-MYC, BCL2 and BCL6 [54].

#### 3.1.3. Oral Squamous Cell Carcinoma (OSCC)

OSCC represents the 10th most common malignancy in women and the 6th most common malignancy in men, with an incidence rate of 84–97% of all oral cancers worldwide [8]. OSCC commonly develops on the tongue, lips and surface of the mouth and is clinically characterized by a red and white or red lesion with a slightly uneven surface and distinct margins. Early-stage lesions may cause discomfort, though are usually painless, but as they progress, they present with ulceration with irregular margins, hard to palpation and nodularity [8,55,56], as shown in Table 1. Risk factors are tobacco and alcohol use, age, gender, toxic habits, intense exposure to sunlight, premalignant lesions, pathological factors [57,58,59], as shown in Table 1. Approximately 50% of OSCC tumors report that a dysfunctional p53 gene plays an important role in apoptosis-inducing mechanisms [8]. The risk of OSCC may be increased by environmental factors such as smoking, irradiation and viral infection and may lead to activation of proto-oncogenes (*Ras*, *MYC*, *EGFR*) or inhibition of tumor suppressor genes (TB53, pRb, p16) [59].

**Table 1:** Oral cancer types, symptoms and risk factors.

Types of Oral Cancer	Incidence	Symptoms	Risk Factors
Oral cancer (OC)	10th most common cancer in the world [1,2,3,4]	Unexplained growths or sores in the mouth, painless or painful lumps, difficulty swallowing, various forms of ulcers [37]	Tobacco, alcohol, chronic inflammation, HPV, immunosuppression, ultraviolet radiation [8,9,10,11]
Oral Squamous Cell Carcinoma (OSCC)	90% of all oral cancers; 6th most common cancer in men worldwide [8]	Red and white or red lesion, ulceration, nodularity [55]	Smoking, alcohol, sunlight exposure, premalignant lesions [55,58,59]
Oral melanoma	Rare (0.2–8% of all melanomas) [8,47]	Brownish-black spot, macule or nodular lesion with various shades of gray, red, purple or areas of depigmentation [8,46,48]	Smoking, alcohol, sun exposure, HPV [46,47]
Lymphoma	The third most common malignancy in the oral cavity [8]	Swelling, pain and paresthesias [53,54]	Immunosuppression, UV radiation, viruses, occupational exposure, chronic autoimmune and inflammatory disorders [52]
Oral verrucous carcinoma (OVC)	5% of squamous cell carcinomas [45]	A painless, grayish-white, warty, painless exophytic mass [60]	Chewing tobacco use, alcohol, poor oral hygiene, HPV, including chronic inflammation [60,61]
Oral Leukoplakia (OL)	2.6% of world population [56,59]	White lesion (homogeneous lesions) or mixed white and red lesion (non-homogeneous lesions) [56,62]	Tobacco, areca nut, alcohol and HPV [63,64]
Oral Erythroplakia (OE)	0.2–0.8% of world population [63]	Solitary red spot [65]	Tobacco, areca nut, alcohol and HPV [63]
Oral Submucosal Fibrosis (OSMF)	>5 million patients globally [66]	Ulcers, xerostomia, submucous fibrosis, burning sensation and a reduced mouth opening [67]	Autoimmunity, vitamin B, C and iron deficiencies, betel nut chewing, spicy food consumption, HPV and genetic mutations, inflammatory factors [66,68]
Oral Lichen Planus (OLP)	2% of the general population [69]	Intermittent bursts and polymorphic clinical features (reticular, erosive, atrophic, plaque, papular, bullous) [70]	Genetic predisposition, microorganisms, mechanical trauma, stress, systemic diseases [71]
Proliferative verrucous Leukoplakia (PVL)	Less than 1% of adults in the United States, no incidence data worldwide [72]	Widespread white lesions with high recurrence rate and high probability of malignant transformation [59]	Smoking, HPV [73]

#### 3.1.4. Oral Verrucous Carcinoma (OVC)

Verrucous carcinoma is a subtype of squamous cell carcinoma that mainly affects the buccal mucosa, followed by the hard palate, buccal floor and gingiva [60]. This type of cancer has a slow onset, accounts for approximately 5% of squamous cell carcinomas, is predominantly found in males, and the median age at diagnosis is between 49 and 69.5 years [45,61]. Clinically, it appears as a painless, greyish-white, warty, cauliflower-like, painless exophytic mass [60]. Known etiologic factors for the development of OVC are inhaled and chewing tobacco use, alcohol consumption and betel nut chewing, chronic inflammation, ulcers, poorly fitted dentures, immunosuppression, poor oral hygiene, HPV, previous mucosal lesions or scarring [60,61]. The most investigated markers in the carcinogenesis of oral verrucous carcinoma are p53, Ki-67, cyclin-B1 and cyclin-D1 [61].

#### 3.1.5. Potentially Malignant Disorders (PMDs)

They are defined as diseases of the oral mucosa, multifocal in nature within the entire upper aerodigestive tract, with a significantly increased rate of occurrence of squamous cell carcinoma compared to apparently normal mucosa [56,73]. This term usefully encompasses both localized lesions and more generalized disease, as shown in Table 1 [63]. Characteristically, OPMD involves different anatomical sites in the oral cavity and presents with various clinical attributes, represented by different sizes, color variations (white, red and mixed white-red), and morphological changes (plaque/plaque, smooth, grooved, wrinkled, granular, atrophic) [73]. The list of mucosal conditions considered potentially malignant includes oral erythroplakia (OE), oral leukoplakia (OL), neoplastic leukoplakia, proliferative verrucous leukoplakia, oral lichen planus (OLP), oral submucous fibrosis (OSMF) and chronic traumatic ulcers. Worldwide, estimates of the prevalence of potentially malignant oral conditions suggest a global figure of between 2 and 3% [57,73]. Approximately 2.6% of the world’s population has leukoplakia and 0.2–0.8% has erythroplakia [45].

Oral leukoplakia typically accounts for between 60 and 70% of all PMD and is described as a white patch that cannot be erased from the mucosal surface and cannot be attributed to any other identifiable pathophysiologic cause. Patients with OL have a prospective risk of malignancy of between 1% and 30% [56,59]. Risk factors associated with OL are tobacco, areca nut, alcohol and HPV. Clinically, OL can be divided into two subtypes: homogeneous and inhomogeneous. Homogeneous lesions are uniformly white, and the surface is flat or slightly wrinkled, while non-homogeneous lesions are more irregular, the surface may be flat, mottled or nodular, with a mixture of white and red color [62,63,64].

Chromosomal deletion in the 3p14 and 9p21 regions in leukoplakia probably increases the invasive potential of oral cancer. Deletion in regions 4q, 8p, 11q and 17p is also observed in leukoplakia [73]. Proliferative verrucous leukoplakia (PVL) is an uncommon variant of oral leukoplakia that occurs in less than 1% of adults in the United States, with a higher incidence among women after the age of 60 years; no worldwide incidence data on proliferative verrucous leukoplakia are reported [63,72]. It clinically presents as an asymptomatic, slowly spreading, white, warty plaque with a high recurrence rate and a high likelihood of malignant transformation [59,63]. The etiology of PVL remains unclear; however, tobacco is excluded as an etiologic factor, as PVL has been observed in both smokers and nonsmokers [73].

The genetic alteration involves a change in ploidy level. Aneuploidy is present in 89.2% of PVL. Loss of heterozygosity (LOH) is the most common alteration in PVL. Allelic loss at 9p21 was present. INFα, D9S1748 and D9S171 are localized to 9p21 loci. Loss of one or more of these markers has been reported in patients with PVL [73]. The clinical appearance of oral erythroplakia is characterized by a single, often well-demarcated, flat, erythematous, bright red, erythematous lesion. The soft palate, buccal floor and buccal mucosa are the most common localizations of OE [63,65]. Oral erythroplakia is associated with tobacco, alcohol and HPV abuse (Table 1), affects middle-aged adults and has a high rate of malignant transformation [63,65]. A high prevalence of p53 mutation is reported in erythroplakia with a frequency of 46%. Genetic alteration, such as polysomy of chromosomes 7 and 17, LOH or allelic upregulation at 9p, 3p, in the Rb, p53 or DCC gene (netrin 1 receptor) region have been implicated in erythroplakia [73].

Oral submucous fibrosis (OSMF) is a precancerous condition of the submucosa that causes inflammation, progressive fibrosis, burning sensation, reduced mouth opening, ulcers, taste disturbance, xerostomia, tongue depapillation, whitening and corky texture of the oral mucosa [67,74,75]. According to World Health Organization (WHO) statistics, there are >5 million patients (Table 1) with OSMF worldwide [66]. Etiologic factors that may lead to the development of OSMF include inflammatory factors, autoimmunity, betel nut chewing, vitamin B, C and iron deficiencies, spicy food consumption, human papilloma virus (HPV) infection and genetic mutations [66,68].

Clinical studies have reported that transforming growth factor-beta (TGF-β), beta fibroblast growth factor-beta (bFGF), connective tissue growth factor (CTGF), tumor necrosis factor-α (TNF-α) alpha-smooth muscle alpha-actin (α-SMA), serum c-reactive protein, ROS levels, matrix metalloproteinases (MMPs) and tissue inhibitors of metalloproteinases (TIMPs) were abnormally expressed in the oral submucous fibrosis (OSMF) group; the frequency of *HLA-A10*, *HLA-B7* and *HLA-DR3* dominance was increased in OSMF patients. In addition, polymorphisms of *COL1A1*, *COL1A2*, *COLase*, *LY oxidase*, *TGF-β1* and *cystatin C* genes are associated with OSMF [67,68].

Oral lichen planus (OLP) is a chronic inflammatory disease whose pathogenesis involves epithelium-directed T-cell mediated inflammation in response to unknown antigen(s). The disease has polymorphic clinical features (reticular, atrophic, plaque, papular, erosive, bullous) evolving in intermittent attacks [70]. The estimated global prevalence of oral lichen planus is about 2%; it is twice as common in women around the age of 50–60 years, but it can also occur in children and young adults [69]. In the pathogenesis of OLP, there are several potential factors involved, such as mechanical trauma, genetic predisposition, stress, insomnia, gingival disorders, nutritional status, systemic diseases, microorganisms, lifestyle, mucosal trauma [71]. Genetic alterations include monosomy of chromosome 9, expression of the tumor suppressor gene p53 concomitant with increased PCNA (proliferating cell nuclear antigen) expression, loss of c-erbB-2 function and increased telomerase activity [76].

### 3.2. Cellular and Molecular Pathway of Pathogenesis

Oral cancer is a multifactorial disease that develops in several stages because of various genetic changes that can occur in the oral mucosa (Table 2). Carcinogenesis is a process involving a genetic or epigenetic damage (Table 2) in susceptible cells that, as a result of the activation of proto-oncogenes or inactivation of tumor suppressor genes or both, undergo clonal expansion [16,59,77]. Activation of proto-oncogenes (*Ras*, *Myc*, *EGFR*) or inhibition of tumor suppressor genes (*TB53*, *pRb*, *p16*) may increase the risk of OSCC [59].

It is known that about 10% of all malignant tumors have a significant genetic component and p53 inactivation is the most common genetic change in all human cancers. Loss of p16 protein has also been observed in the most advanced pre-malignant lesions. Loss of chromosome 17p is also common in most human cancers, including OC [78,79]. Tumor development and progression result from the accumulation of lesions in multiple pathways, leading to the triggering of a sequence of events such as DNA damage, cell hyperproliferation, apoptosis, angiogenesis, invasion of surrounding tissues and distant metastasis [16,80].

#### 3.2.1. Apoptosis

Apoptosis is an evolutionarily conserved, energy-dependent, biochemically mediated process of cell death responsible for the elimination of infected or transformed cells, maintenance of homeostasis and normal cell turnover in the body [81,82,83]. Apoptosis can be induced in cancer cells through two different pathways, intrinsic and extrinsic, in which the signals that initiate cell death originate from inside or outside the cell, respectively [81,84]. The extrinsic pathway was regulated by the death receptor containing Fas receptor, tumor necrosis factor (TNF) receptors and TNF-related apoptosis-inducing ligand (TRIAL) receptors. These receptors bind to extrinsic ligands and the shared intercellular information is disrupted, and several caspases (cysteine-aspartic protease—which controls the whole process of apoptosis by signaling, initiating and regulating processes) like proteases affect cellular function, and this disruption of cell regulatory function leads to cellular death [82,85].

The intrinsic pathway is activated when the cell is subjected to stress from within due to various factors, such as DNA damage following exposure to X-rays or UV, hypoxia, chemotherapeutic agents, accumulation of misfolded proteins inside the cell [86]. The critical step of intrinsic apoptosis is the activation of pro-apoptotic effectors of the BCL2 family, BAX, BAK and possibly BOK, which cause outer membrane permeabilization (MOMP) and commit cell death. MOMP leads to the release of pro-apoptotic proteins, including CYCS and SMAC, from the mitochondrial intermembrane into the cytosol. CYCS assembles with APAF1, dATP and pro-CASP9 in the apoptosome, leading to the activation of CASP9, which, in turn, promotes the activation of the caspases CASP3 and CASP7. The activation of the caspases is facilitated by SMACs, which sequester and/or degrade IAP family members that inhibit apoptosis [82,87].

#### 3.2.2. Tumor Angiogenesis

It represents a complex and dynamic physiologic process involving the formation of new blood vessels, with a physiologic or pathologic effect [24,88]. It is a fundamental step that plays a crucial role in the phases of tumor growth, invasion and metastasis. With the advances in cellular and molecular biology, various biomolecules involved in tumor angiogenesis have been identified, such as vascular endothelial growth factor (VEGF), platelet-derived growth factor (PDGF), fibroblast growth factor 2 (FGF-2), chemokines, ephkrine, apelin (APLN) [89,90,91,92]. VEGF induces diverse processes such as migration, vasodilation, survival, permeability, proliferation, cell adhesion and vascular shape through different signaling pathways in endothelial cells by binding to the VEGF receptor-2 (VEGFR-2). These are as follows: phospholipase-Cγ (PLC-γ)/protein kinase C (PKC); p38-mitoogen-activated protein kinase (MAPK); phosphoinositide-3-kinase (PI3K)/protein kinase B (PKB); SRC and FAK [89,93,94,95]. In normal cells, the anti-VEGF enzyme protein kinase G (PKG) limits beta-catenin, which solicits angiogenesis. In cancer cells, it has been found that cancer cells no longer produce PKG [27].

#### 3.2.3. Metastasis

Metastasis of cancer cells is a complex process involving various cytophysiological changes by which cancer cells escape from the primary tumor, enter the blood or lymphatic vessels and develop new tumors in other parts of the body. A variety of cells and molecular components surrounding the tumor provide signals that enhance the metastatic potential of cancer cells [96,97]. Cancer progression depends largely on the proteolytic activity of numerous matrix metalloproteinases (MMPs), able to degrade all components of the extracellular matrix (ECM), which release cytokines bound to the cell surface, matrikines [98,99]. The proteolytic activity of MMPs is modulated by tissue inhibitors of metalloproteinase (TIMP). A balance between MMP activity and TIMP levels is required for the maintenance of tissue homeostasis but can be chronically disrupted in pathological processes [100,101].

#### 3.2.4. Signaling Pathways in Oral Cancer

Understanding the molecular mechanisms underlying these pathways is essential for the identification of potential therapeutic targets, enabling the development of more effective and precise treatments for oral cancer [102,103,104].

- EGFR (epidermal growth factor receptor) pathway

EGFR is a surface tyrosine kinase receptor of the ErbB family. This protein plays a key role in numerous processes affecting tumor growth, differentiation, progression, invasion, metastasis and inhibition of apoptosis [105]. EGFR is highly expressed in several carcinomas, including OSCC. EGF binds to the extracellular domain of EGFR and induces its dimerization; then, intrinsic kinase activity is activated. Subsequently, the intracytoplasmic domain of EGFR is autophosphorylated at several tyrosine residues. These phosphorylated tyrosine residues are bound to several adaptor proteins, and the EGF/EGFR signaling pathway is activated, which plays a critical role in cancer cell proliferation [106].

Thus, the EGF/EGFR signaling pathway may represent an important molecular target in cancer therapy. Some informative studies report the efficacy of EGFR-targeted therapies on cancer progression using EGFR tyrosine kinase kinase inhibitors (TKIs) or antibodies that neutralize EGFR [105,106].

- PI3K/AKT/mTOR pathway

The PI3K/AKT/mTOR (PAM) signaling pathway is a highly conserved signal transduction network in eukaryotic cells that promotes cell survival, cell growth, metastasis and cell cycle progression [107]. Signaling from growth factors to transcription factors in the PAM axis is tightly regulated by multiple cross-interactions with several other signaling pathways, and deregulation of signal transduction may predispose to cancer development [99,108]. Current therapeutic options are insufficient for patients, and tumor complexity and heterogeneity require personalized drugs or targeted therapy [109,110].

- Wnt/β-Catenin pathway

WNT/β-catenin signaling directs diverse physiological processes including embryonic development, tissue homeostasis, growth and regeneration processes. Abnormal WNT/β-catenin signaling is associated with the occurrence of various cancers [111,112]. The Wnt ligand/receptor interface, the TCF/β-catenin transcription complex and the β-catenin destruction complex are key elements targeted in preclinical and clinical evaluations. Thus, opportunities will be created for clinicians to develop more effective and targeted remedies for cancer patients with aberrant Wnt/β-catenin signaling [113,114].

- TGF-β (transforming growth factor beta) pathway

Transforming growth factor β (TGF-β), a crucial cytokine, with complex and essential roles by contributing to fibrosis by promoting extracellular matrix deposition and tumor progression by inducing epithelial–mesenchymal transition, immune suppression and neovascularization in advanced stage cancer. This signal controls multiple cellular responses during embryonic development and tissue homeostasis through canonical and/or non-canonical signaling pathways [115,116,117].

- NF-κB pathway

Nuclear factor-kappa factor B (NF-κB) is a key nuclear transcription factor named for its specific binding to the nuclear factor-κB (GGGACTTTCC) sequence in the enhancer region of the immunoglobulin kappa chain gene, which regulates processes in tumor promotion. Recent evidence shows that inflammatory microenvironment is associated with tumor migration, invasion and metastasis. Hence, tumor necrosis factor-α (TNF-α) plays an essential role in regulating the inflammatory process in tumor development [118,119].

The NF-κB pathway may represent a promising therapeutic target by suppressing NF-κB in myeloid cells or tumor cells, ultimately installing tumor regression. The components of the NF-κB pathway [120] need to be carefully evaluated and selected in order to design targeted therapies due to its vital role in diverse biological activities (Table 2).

**Table 2:** Genetics and epigenetics of oral pathology.

Oral Pathology	Genetics/Epigenetics	References
Oral cancer (OC)	*VAV2*, *IQGAP1* as the genetic basis of oral cancer in a family;	[121]
Oral squamous cell carcinoma (OSCC)	Activation (*RAS*, *MYC*, *EGFR*) Biomarkers: G3BP1, B7-H6 and FAM3C for prognosis; MIA2 expression; miRNA DNA methylation abnormalities	[55,59,122,123,124]
Oral melanoma	BRAF, NRAS mutations	[49]
Lymphoma	*BCL2*, *BCL6*, *MYC* mutations	[125]
Oral verrucous carcinoma (OVC)	*CTTN*, *FOLR3*, *ORAOV1*, *ORAOV1*, *PPFIA1* and *RNF121* genes with poor prognosis in oral verrucous hyperplasia regions with copy number variation: 1p35, 1p36, 11q13, 6p21, 14q22 and 22q12; Most investigated markers: p53, Ki-67, cyclin-B1, cyclin-D1, p21, p27; 8 genes may determine the differences in identity of OVC two OSCC cancers (ADAMTS12, COL4A1, COL4A2, INHBA, MMP1, SERPINE1, TGFBI, HLF)	[60,73,124,126,127,128]
Oral Leukoplakia (OL)	Chromosomal deletions (3p14, 9p21), (4q, 8p, 11q, 17p; 14 essential genes; signal transducer and activator of transcription 5B (STAT5B) and epidermal growth factor receptor (EGFR) and eight miRs, such as miR-549, miR-205 and miR-2;	[129]
Oral Erythroplakia (OE)	Polysomy of chromosomes 7 and 17, loss of heterozygosity (LOH), allelic gain in genes 9p, 3p, Rb, p53, DCC	[73]
Oral submucosal fibrosis (OSMF)	genetic susceptibility (collagen nucleotide polymorphism, MMPs, TIMPs, TGF-β1, CST3 and LOX)	[130]
Oral Lichen Plan Oral (OLP)	*P53*, *PCNA* mutations, loss of C-ERBB2 function	[76]
Proliferative verrucous leukoplakia (PVL)	Aneuploidy; LOH at 9p21; loss of INFα, D9S1748, D9S171 markers; loss of p14 ARF; p53 positive	[73]

The latest research over genetic (mutations, polymorphisms, allelic activation or loss of heterozygosity) and epigenetic (miRs) causes of oral cancer different forms reveal many mechanisms (Table 2) that vary from one category to another. Most common studies related to genetic causes of this type of pathology have been analyzed for OVC, identifying specific genes. All these genetic and epigenetic influences contribute significantly to understanding the analyzed pathologies’ complexity, thus providing a reasoned support for personalized therapy.

### 3.3. Oral Cancer Incidence, Mortality and Survival

Oral cavity and pharyngeal cancers are grouped together in global reports [131,132,133]. According to their incidence, the eight different forms of oral cancer are categorized as follows: buccal mucosa (32%), tongue (22%), lower lip (11%), palate (11%), vestibule (8%), alveolus (5%), floor of the mouth (5%) and gingiva (3%) [132,134]. There is a high prevalence of OC in Asian countries in the south–central region, including Sri Lanka, India and Pakistan, with India accounting for more than 1/3 of patients (135,929) and 1/5 of deaths (75,290) [133,134]. According to data collected by the Global Cancer Observatory (GCO), there were 377.713 cases of OSCC worldwide in 2020, with the majority of these occurring in Asia [55,135]. Men and women have roughly identical incidence and mortality rates in Japan, Indonesia, Mexico, Mexico, India, Bangladesh and Pakistan [31]. Racially, black Americans are more likely to develop oral and pharyngeal cancer than white Americans [38]. The GLOBOCAN reports show that the incidence rate and age-standardized incidence rate of oral cancer are highest in more prosperous countries, while the mortality rate is more frequent in less developed regions, indicating social inequality [34,133]. Nearly 95% of cases occur in people over 40 years of age, who are on average 65 years old [38]. The incidence rate of this pathology in young men and women has increased significantly over the last 25 years, but the mortality rate has decreased only slightly [136]. Mortality rates are influenced by the incidence of oral cancer, access to treatment and variations in the distribution of areas, it can be recorded between 1 and 15 per 100,000 people in different regions and exceed 10 per 100,000 in Eastern European countries such as the Czech Republic, Hungary and the Slovak Republic [9].

This situation has failed steadily in France since peaking in the early 1990s, and the decline correlates with a reduction in per capita alcohol consumption. Mortality rates have fallen steadily in Australia and Hong Kong SAR, China, but have increased in Japan and the Republic of Korea. In the United States, the five-year survival rate improved by more than 11 percentage points between 1992 and 2006 and is now about 65%. In Europe, it is about 50%. In India, five-year survival is lower than 35%; in China, the Republic of Korea, Pakistan, Singapore and Thailand, it varies between 32 and 54%. In general, the five-year survival for early localized cancers exceeds 80% and falls to less than 20% when regional lymph nodes are involved [9].

### 3.4. Etiology and Risk Factors

Risk factors involve anything that significantly increases a person’s chance of developing a disease, such as oral cancer [137]. The most important etiologic factors noted for oral cancer induction are as follows: tobacco use, chewing betel quid/Qat, excessive consumption of certain hot beverages such as mate, alcohol consumption, a diet lacking in fresh fruits and vegetables, infectious agents (*Candida*, viruses, human papillomavirus (HPV), poor oral hygiene, immunosuppression and, in the case of lip cancer, sun exposure, genetic alterations [138,139,140].

#### 3.4.1. Smokeless and Smoking Tobacco

Cigarettes and other burned tobacco products are dangerous nicotine delivery devices that contain a complex mixture of toxic substances that may be tumor promoters and co-carcinogens. Consistent evidence from numerous studies indicates that tobacco smoking in any form increases the risk of oral cancer by two to ten times in men and women. The risk increases substantially with duration (more than 20 years) and frequency of tobacco use (daily more than 20 cigarettes) [56,141]. Smokeless tobacco (SLT), an umbrella term that includes tobacco marketed for oral (chewed, mouthed, sucked, dipped) or nasal use, contains varying amounts of nicotine and nitrosamines. SLT use has already been integrated as an independent risk factor for the development of OC in numerous studies [141,142]. Areca nut (AN) chewing is one of the main risk factors for the development of oral cancer in Southeast Asia. In Taiwan, for example, about 85% of all oral cancer patients show an association with this habit [143,144]. Recent studies based on animal models and in vitro experiments show cellular and molecular effects induced by AN. These include promotion of epithelial–mesenchymal transition, initiation of autophagy, genotoxicity, tissue hypoxia, cytotoxicity and cell death [145,146]. The consumption of Qat and mate is thought to have an important impact on the development of oral cancer in humans. A major role in the enhancement of human oral carcinogenesis could also be represented by the methods of cultivation and preparation of Qat and mate, respectively [147].

#### 3.4.2. Dietary Factors

Several dietary nutrients with specific mechanisms of action may help reduce the risk of developing oral cancer. For oral and pharyngeal cancers in general, the consumption of more fruits, vegetables and related micronutrients, such as vitamin C and folate, is associated with a lower risk of cancer. This benefit is also supported by the presence of vitamins with antioxidant, antiproliferative, immunomodulatory properties [148,149]. Chronic inflammation plays a key role in the development of some oral and pharyngeal cancers. The tumor microenvironment is connected to different stages of tumorigenesis and composed of different cell types, such as fibroblasts, myofibroblasts, adipose cells, immune and inflammatory cells and extracellular matrix (ECM) [138].

Consumption of alcohol, such as ethanol, is known to be one of the etiologic factors in the onset and development of OSCC. Acetaldehyde, the metabolite of ethanol, can cause DNA damage and block DNA synthesis and repair, while both ethanol and acetaldehyde can disrupt DNA methylation. Ethanol can also induce inflammation and oxidative stress, leading to lipid peroxidation and further DNA damage [150]. Although the period of exposure to alcohol is short, it appears to increase the permeability of the oral mucosa to potential carcinogens as well as to tobacco-specific nitrosamines [151]. Drink Mate, a leaf infusion commonly consumed several times a day, especially in South America, is usually drunk very hot. Studies suggest that drinking mate may slightly increase the risk of oral cancer [9].

#### 3.4.3. Oral Infections

Of all *Candida* species, *Candida albicans* has the highest prevalence in the development of OSCC [15] and is considered to be one of the most frequently researched oral pathogens in this pathology [152,153]. *Candida albicans* up-regulates oncogenes, potentiates a premalignant phenotype, and is implicated in the stages of malignant promotion and progression of oral cancer [154]. HPV-positive OSCC shows one of the fastest evolutions of all cancers in high-income countries, with the prevalence of HPV-associated oral cancers increasing significantly over the years [155,156,157]. Infection of the oral squamous epithelium with HPV could lead to either a tight productive life cycle associated with progressive differentiation of epithelial cells, culminating in the generation and shedding of virions, or a transformative life cycle leading to the transformation of differentiated growth-arrested cells into actively proliferating cells [157].

#### 3.4.4. Immunosuppression

Immunosuppressed people are more likely to develop oral cancers. Patients infected with the human immunodeficiency virus (HIV) are prone to developing lymphomas but not OSCC. Immunosuppressed organ transplant patients have been shown to develop lip cancers as a result of high exposure to risk factors such as solar radiation and smoking [158].

### 3.5. Treatment

Management of OSCC involves a multidisciplinary approach. The primary treatment option for non-metastatic OSCC is ideally surgery, and in the early stages of the disease to minimize surgery-related morbidity, less invasive curative surgical approaches are preferred [159]. Besides surgery, the main treatments for oral cancer and/or adjuvant therapy are radiotherapy and chemotherapy [16,45]. Treatment options vary depending on factors such as tumor location, age and functional outcome. The most commonly used chemotherapeutic drugs in patients with oral cancer are paclitaxel, 5-fluorouracil, cisplatin, doxorubicin and docetaxel and inhibitors such as bevacizumab, cetuximab and erlotinib [31,160].

The most potent and commonly used first-line treatment for recurrent or metastatic oral cancer, with superior survival benefit, is the combination of cetuximab and platinum-based chemotherapy involving cisplatin or carboplatin [161,162]. Although many oral cancer patients are successfully treated with adjuvant therapy or anti-cancer drugs, these treatments are highly toxic to normal cells and tissues [16]. Undesirable effects that can lead to cancer treatment failure because of chemo- and radiotherapy treatments include changes in taste, stomatitis, xerostomia, mucosal ulceration, discomfort, nausea, vomiting, diarrhea, anorexia and increased salivary viscosity, fatigue and hair loss [16,163,164,165]. In addition, besides the risk of toxicity, the financial burden of these treatments on patients limits their use [166]. The main disadvantages of chemotherapy that affect the patient’s survival rate and quality of life are the possibility of cancer recurrence, the development of drug resistance, and unintended lethal effects on tissues [32].

#### 3.5.1. Promising Therapeutic Alternatives

Therefore, there is a challenge on the use of alternative or additional therapy for cancer treatment to limit the occurrence of side effects. Recent studies have shown chemopreventive effects of natural products against oral cancer cells. Many bioactive compounds derived from natural products have demonstrated anticarcinogenic potential due to their high cytotoxicity on cancer cells and low toxicity on normal cells [167,168]. Therefore, every year, more and more novel cytotoxic compounds identified and isolated from plants or their synthetic derivatives constitute new possibilities for integration into cancer treatments [169,170]. The most common herbal anticancer drugs are vincristine, naringin, paclitaxel, vinca alkaloids, etoposide, taxanes derivates, podophyllotoxin, camptothecin derivatives and roscovitine. They are made with a variety of organic ingredients, including whole grains, fruits, nuts, vegetables and herbs [31,171]. Phytochemicals have attracted particular interest from researchers because they occur naturally in foods and usually have multiple benefits [172].

“Phyto” in the word phytochemicals comes from the Greek word *phyto*, meaning plant. Phytochemicals are therefore the chemicals in plants. It is estimated that more than 5000 phytochemicals have been identified and that there are more than 150,000 edible plants on earth, but a large percentage still remains unknown and needs to be identified [173]. In numerous studies, medicinal plants and their extracts have been shown to possess potent antioxidant, anti-inflammatory and anticarcinogenic properties that exhibit diverse mechanisms of action that slow cell proliferation and the rate of malignant transformation of various oral cancers such as oral leukoplakia, oral submucous fibrosis and oral lichen planus [174].

Phytochemicals show variations in their solvent affinity and heat tolerance. Solvent selection also affects the quality of recovered phytochemicals and their application in food and nutraceutical development [21]. In general, many studies highlight that polyphenol (epigallocatechin-3-gallate, curcumin, resveratrol), alkaloids (piperine), carotenoids (astaxanthin), flavonoids (genistein and quercetin), probiotic molecules (bacteriocin and extracellular polysaccharides) and marine peptides could form the basis of natural therapeutic options with promising effect for the treatment of oral cancer cells [12,175].

These natural compounds fall into various categories of primary and secondary metabolites used to treat a growing number of conditions. Plants and plant-derived products represent a revolutionary field as they are simple, safer, environmentally friendly, involve low cost and are less toxic compared to conventional treatment methods [176,177,178].

After analyzing the information about the plants with possible applications in oral cancer, a tendency was found to target the molecular mechanisms, especially those associated with apoptosis (Table 3).

#### 3.5.2. Oral Cancer Chemoprevention

Chemoprevention is defined as the administration of one or more synthetic or natural compounds to control, slow, reverse or suppress carcinogenesis [210,211]. Chemoprevention also involves prevention of premalignant lesions such as preinvasive neoplasia, intraepithelial neoplasia or dysplasia, depending on the organ system. Therefore, phytochemicals can be considered as chemopreventive agents with a beneficial role in the prevention of all specific aspects of carcinogenesis, aimed at reducing the chances of cancer occurrence and reducing the morbidity rate [210,211,212].

An ideal chemopreventive agent should have excellent bioavailability at the target site, minimal or no toxicity, high multisite efficacy, low cost and human acceptability [211]. There are three levels of oral cancer chemoprevention: primary, secondary and tertiary [213,214]. Primary prevention takes the form of controlling and/or eliminating risk factors for cancer, such as smoking, chewing betel quid, excessive alcohol consumption, prolonged sun exposure and poor diet. The aim of the primary preventive measure is to educate and counsel patients about the potential harmful effects of risk factors associated with oral cancer development [33,210].

Secondary prevention aims at early detection and diagnosis of premalignant lesions (e.g., oral leukoplakia) through advanced screening techniques and intervention measures to prevent progression to malignancy [210,213]. Tertiary prevention is indicated when a person has previously had cancer or oral cancer, when a person is immunosuppressed or has GVHD due to stem cell transplantation. Assessment of genetic mutations, search for personalized genetic recommendations and estimation of personal cancer risk are included in this stage [30,215]. Studies to date have applied oral cancer chemoprevention at the secondary and tertiary levels [33]. The rapid understanding of the molecular mechanism of oral carcinogenesis offers new promise for innovation in the development of safe and effective chemopreventive agents that can be easily administered to populations at high risk of oral cancer [216,217,218].

## 4. The Role of Phytochemicals in Oral Cancer Treatment

### 4.1. Mechanisms of Action of Plants and Their Bioactive Compounds

Research based on the mechanism of action of natural plant-derived compounds as anticancer agents provides an important alternative approach that can be effectively used to prevent, cure and minimize the side effects of cancer therapies.

The importance of determining a definitive strategy for exploring novel highly selective compounds for OSCC is suggested by the presence of multiple types of cell death [219]. In several studies phytochemical compounds present in natural plants and their extracts have been shown to exhibit a variety of biological activities (Table 3) including antioxidant, anti-inflammatory, antibacterial, antiproliferative, regulating the cell cycle, proapoptotic, antiinvasive, antiangiogenic, antimetastatic, protecting normal cells from reactive oxygen species (ROS) properties, having a key role in chemoprevention [189,220,221].

**Figure 1:**
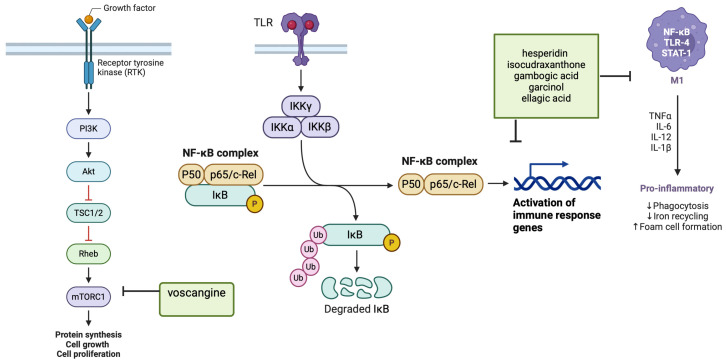
Interaction mechanism between a selection of plant bio-compounds and molecular pathways involved in pro-inflammatory inhibition (Created in BioRender. Schroder, V. (2024), BioRender.com/j83o028 (accessed on 16 October 2024)).

The antioxidant defense mechanism of phytochemicals enhances specific genes encoding antioxidant proteins via the key transcription factor (Figure 1), and its downstream antioxidant proteins include heme oxygenase-1, catalase, glutathione peroxidase, quinone oxidoreductase 1, superoxide dismutase, glutathione S-transferase and glutamate cysteine ligase. Finally, the anti-inflammatory mechanism of phytochemicals is mediated by the inhibition of the activity of several pro-inflammatory cytokines, including TNFα, IL-1, IL-6, iNOS, COX-2 and nuclear factor-κB (NF-κB), (Table 4) [169,222,223,224].

Phytochemicals modulate coding RNAs as well as non-coding RNAs such as microRNAs and short non-coding RNAs [225]. Phytochemicals, such as those found in cranberries and blueberries, by inhibiting key pathways involved in carcinogenesis, such as the NF-κB pathway, the MAP kinase kinase pathway and PI3K/AKT/mTOR signaling, (Figure 1) have been shown to have a promising effect in anticancer therapy [226,227].

The exertion of antineoplastic effects of bioactive phytochemicals as those presented in Tabel 4, for exemple 6-Shogaol (ginger), capsaicin (chili pepper), latifolin (*Dalbergia odorifera* T. Chen), rosmarinic acid is achieved by modulating various signaling pathways and triggering apoptosis [218,228,229,230,231,232]. The signaling pathways mainly associated with cancer are the mitogen-activated protein kinase (MAPK) pathway, the nuclear factor kappa B (NF-kB) pathway and the transcription-activating protein (STAT) pathway [228]. For ellagic acid analogs, gambogic acid (GA), isocudraxanthone K (IK), Toll-like receptors, epidermal growth factor receptor, cytokine receptors, and tumor necrosis factor receptor are specifically involved, altering the expression of downstream genes and proteins [218,233,234].

Some recent studies highlight that pinosylvin reduced MMP-2 activity and expression while increasing TIMP-2 levels and also decreased phosphorylation of ERK1/2 protein in oral SCC-9, SAS and HSC-3 human cancer cells. This organic compound suppresses the metastatic potential of oral squamous cell carcinoma, thus being a possible therapeutic candidate for the prevention of oral cancer metastasis [235,236].

Another study reveals that Garcinone E, a phytocompound from *Garcinia mangostana* L., disrupts the fundamental processes that allow cancer to thrive by significantly reducing the proliferation and colony-forming capacity of HSC-4 cancer cells [237]. In addition, also epigallocatechin-3-gallate (EGCG), pterostilbene, luteolin effectively suppresses cell migration and invasion, two critical factors in cancer metastasis, by decreasing the expression of MMP-2 and MMP-9; modulates cytokine levels, providing a potential new avenue for treating oral cancer [45,237,238,239].

**Figure 2:**
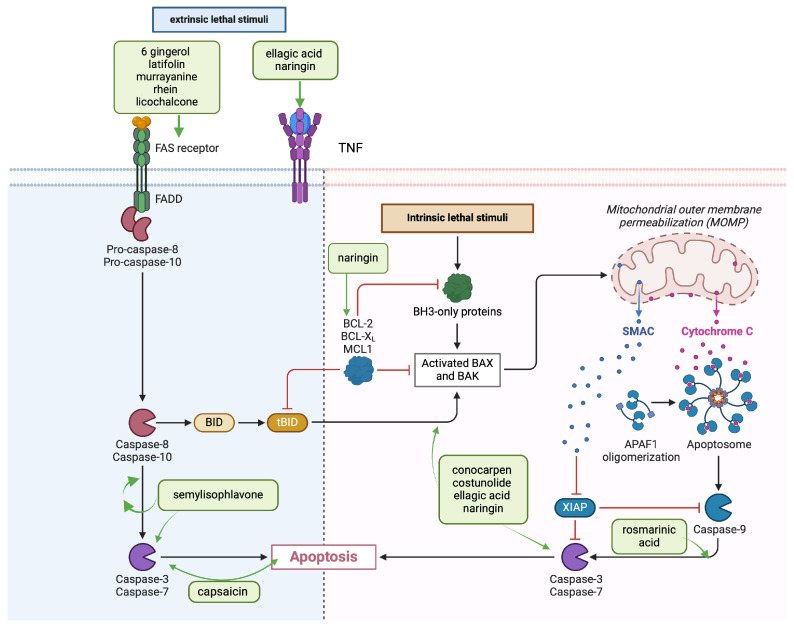
Apoptosis pathways and interactions with plant bio-compounds (Created in BioRender. Schroder, V. (2024), BioRender.com/j83o028 (accessed on 18 October 2024)).

Studies performed on [6]-Gingerol, a bioactive compound from ginger, have demonstrated significant inhibitory effects, influencing the growth of oral cancer cells (Table 4), (Figure 2), by inducing apoptosis. In one study, to evaluate the effects of [6]-gingerol, a compound extracted from ginger, on human oral cancer cell growth, YD10B and Ca9-22 cells were exposed to different concentrations (0, 50, 100, or 150 μM) of [6]-gingerol for 48 h. It significantly inhibited the growth of oral cancer cells by inducing apoptosis and cell cycle arrest in G2/M phase, and inhibited the migration and invasion of oral cancer cells by upregulating E-cadherin and downregulating N-cadherin and vimentin. In addition, [6]-gingerol induced AMPK activation and suppressed the AKT/mTOR signaling pathway [1]. Another study demonstrated that luteolin-7-O-glucoside significantly reduces proliferation, migration and invasion of oral FaDu, HSC-3 and Ca9-22 cancer cells by exerting antimetastatic effects by inhibiting p38 phosphorylation and decreasing MMP-2 expression. The solution (100 mM) was prepared using dimethyl sulfoxide (DMSO) and stored at −20 °C. The DMSO concentration was lower than 0.2% for each experiment. For luteolin-7-O-glucoside treatments, appropriate amounts of stock solution were administered to the medium to obtain the final experimental doses [232].

All these studies not only suggest that phytochemistry may have potential in oral cancer therapy, but also demonstrate that it may present a promising strategy for the discovery of novel drugs against oral cancer [8,16,189]. Phytochemicals demonstrate promising effects including in clinical trials by reducing cell growth, oral lesion size, pain threshold, and new lesion development [218]. In terms of cancer management modalities, these substances can be used in a versatile manner as chemopreventive, chemotherapeutic or chemosensitizing agents [169].

**Table 4:** Phytochemicals in oral cancer treatment: mechanisms and effects.

Mechanism and Molecular Targets	Phytochemical Compounds (PubChem Compound CID) https://pubchem.ncbi.nlm.nih.gov (accessed on 16 October 2024)	Significant Effects Identified and Perspectives
Antioxidant Pro-inflammatory Cytokines inhibitions TNF-α IL-2 IL-6 iNOS COX-2 NF-kB	Berry-derived phytochemicals (bilberries-*Vaccinium myrtillus* L., cranberries *Vaccinium macrocarpum* L. and blueberries (*Vaccinium corymbosum* L.)	Inhibition of inflammation; modulation of apoptosis; reduction in resistance by interfering with the expression of detoxification enzymes; antiproliferative effects in OC [227].
	Cudraxanthone H (CH) (*Cudrania tricuspidata* Bureau) Compound CID: 10449043	Inhibits cell proliferation; apoptosis in OSCC cells, as evidenced by an increased percentage of cells in the sub-G1 phase of the cell cycle, annexin V-positive/propidium iodide-negative cells, and nuclear morphology; new chemotherapeutic agent targeting NF-κb and PIN1 in oral cancer [240].
	Curcumin Compound CID: 969516	Selective cytotoxicity against OSCC cells; showed cell-cycle arrest, by increasing the expression of cyclin-dependent kinase inhibitors (CDKIs) such as p21, p27, p16 or p53 and Rb (retinoblastoma protein); reduce DNA damage on oral cancer cells by inhibition of H2A.X protein expression; induces autophagy, reduces reactive oxygen species, increases intracellular glutathione activity and decreases mitochondrial membrane potential in cancer cells; potential as a therapeutic agent for oral cancer [241].
	Ellagic acid analogs Compound CID: 5281855	Apoptosis in oral cancer cells by activating pro-apoptotic genes; using in silico approaches, the ellagic acid analogues were identified, as a novel, potent and selective CK2 inhibitor [242].
	Gambogic acid (GA) (from traditional Chinese medicine)	Apoptosis and inhibits the NF-κb pathway, reducing cell proliferation in oral cancer cells [233].
	Garcinol (*Garcinia indica G.*) Compound CID: 5490884	Anti-proliferative, pro-apoptotic, cell-cycle regulatory and anti-angiogenic effects on oral cancer cells through inhibition of NF-κB and COX-2; chemopreventive or chemotherapeutic agent for the treatment of oral cancer [243].
	Garcinone E Compound CID: 10298511	It reduces proliferation, suppresses migration and invasion in OC and modulates the immune response. Inhibit metastasis of an oral cancer cell line by blocking invasion, migration and MMP production [237].
	Hesperidin Compound CID: 10621	Reduction in TNF-α, IL-1-β, IL-6, NF-κB, and Bcl-2 mRNA expression levels, highlighting its inhibitory role in cell proliferation, migration, and inflammation processes; significant inhibition of cell proliferation; potential to contribute to more effective treatment strategies [34].
	Isocudraxanthone K (IK) (*Cudrania tricuspidata* Bureau) 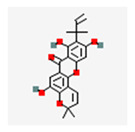 Compound CID: 44426653	Exhibits dose- and time-dependent antiproliferative and pro-apoptotic effects through mitochondria/death receptor, MAPK, NF-*κ*B, and HIF-1*α* signaling pathways; potential candidate for oral cancer chemotherapy [234].
	Naringin 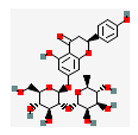 Compound CID: 442428	Promotes apoptosis; down-regulates pro-survival genes; inhibits migration; reduces cell viability in oral cancer cells [31].
	Plumbagina (*Plumbago zeylanica* L.)	Reduces cell viability; induces apoptosis by generating ROS, leading to mitochondrial dysfunction and endoplasmic reticulum (ER) stress; shows promising results for drug-resistant oral cancer [244].
	Semylisophlavone B (*Glycyrrhiza* species)	Induces G2/M cell cycle arrest, apoptosis; inhibits the Ras/Raf/MEK/ERK pathway; potent anticancer agent with potential clinical application in the management of OSCC [245].
MAPK signaling pathways JNK1/2, p38 MAPK PI3K/Akt, GSK-3β, HOTAIR AKT/mTOR, ROS Topoisomerase II,	[6]-Gingerol (ginger) Compound CID: 442793	Apoptosis, causing cell cycle arrest in G2/M phase and suppression of oral cancer progression; reduction in cancer cell migration and invasion by upregulation of E-cadherin and downregulation of N-cadherin and vimentin; therapeutic agent for treating oral cancer [1].
	Erianin Compound CID: 356759	Exerted its cytotoxic effect by inducing cell cycle G2/M-phase arrest and caspase-dependent apoptotic pathways; apoptosis and autophagy; effective anticancer agent for the treatment of oral cancer [246].
	Magnolol (*Magnolia officinalis* Rehder & Wilson) Compound CID: 72300	Induces oral cancer cell apoptosis by increasing the cleavage of caspase and PARP [247].
	Neem *(Azadirachta indica* A. Juss)	Induces autophagy; switches to apoptosis by down-regulating PI3K/Akt; activates GSK-3β, which inhibits autophagy [248].
	Rhein (rhubarb) Compound CID: 10168	Inhibits cell growth by inducing apoptosis and causing cell cycle arrest in S-phase; exhibits significant anticarcinogenic effects both in vitro and in vivo for oral cancer [249].
	Rhinacanthine-C (*Rhinacanthus nasutus* kurz) Compound CID: 6474554	Induces S-phase arrest and apoptosis by activating caspases; inhibits OSCC cell growth; potential as a novel natural anticancer agent or as an adjuvant therapy for oral cancer treatment [250].
	Voacangine Compound CID: 73255	Induces ROS-triggered apoptosis, G2/M cell cycle arrest; inhibits PI3K/AKT pathway; inhibits proliferation of oral cancer cells [251].
Apoptosis Intrinsic and extrinsic signaling Bax, Bcl-2, Cleaved Bax, cMyc BAK, Caspase 3/6/9 FAK/Src, PI3K/AKT/mTOR/p70S6K FasL, Caspase-8, JAK2/STAT3	6-Shogaol (ginger) Compound CID: 5281794	Apoptosis, suppresses EMT by modulating E-cadherin and N-cadherin; inhibits OSCC growth, colony formation, migration and invasion [229].
	Aloe-emodin (*Rheum undulatum* L.) Compound CID: 10207	Inhibits cell proliferation; apoptosis; anti-cancer effects against oral cancer cells [252].
	Brucine Compound CID: 442021	Induces cytotoxicity; promotes oxidative stress, lipid peroxidation, apoptosis, inhibits metastasis in oral cancer cells; significant potential as a significant anticancer agent against oral cancer [253].
	Capsaicin (chili pepper) Compound CID: 1548943	anti-proliferative effects; apoptosis and cell cycle arrest in G1 phase. Throughout the G1- stage, growth-dependent cyclin-dependent kinase (CDK) activity boosts DNA replication and initiates transition of the G1- to S- phase [230].
	Conocarpan(*Piper rivinoides* Kunth) Compound CID: 6474521	Time-dependent cytotoxic effects against OSCC cell lines; apoptosis; canocarpan shows highest selectivity; promising in OSCC cells [254].
	Costunolide (CE) (*Lycium shawii* Roem. & Schult) Compound CID: 5281437	Significantly reduced the expression of the pro-metastatic MMP-2 gene; the expression of the COX-2 gene was significantly reduced in the treated cells; high cytotoxicity against OSCC cells with low toxicity to normal cells; apoptosis, up-regulation of apoptotic genes; down-regulation of anti-apoptotic, metastatic and pro-inflammatory genes [255].
	Latifolin (*Dalbergia odorifera* T. Chen) Compound CID: 340211	Anti-metastatic properties; inhibits cell proliferation, invasion, migration and adhesion; induces apoptosis; making it a promising therapeutic candidate for OSCC [231].
	Licochalcone A (licorice root) Compound CID: 5318998	Induces apoptosis via the FasL signaling pathway, which involves a caspase-dependent FasL-mediated death receptor pathway; reduces cell viability, showing potential as a chemotherapeutic agent for oral cancer [256].
	Licochalcone C (*Glycyrrhiza glabra* L.) Compound CID: 9840805	Inhibits cell viability; disrupts mitochondrial function and suppresses tumor growth by targeting the JAK2/STAT3 signaling pathway; induced the death receptor (DR)4 and DR5 expression level with the generation of reactive oxygen species and the upregulation of CHOP protein expression; promising therapeutic agent for the treatment of oral cancer [257].
	Murrayanine Compound CID: 96942	Induces apoptosis, inhibits the AKT/mTOR and Raf/MEK/ERK pathways; exhibits selective in vivo anticancer effects against oral cancer [258].
	Rosmarinic acid Compound CID: 5281792	Inhibits the growth, spread and proliferation of oral cancer cells by inducing apoptosis and causing G2/M cell cycle arrest [232].
CELL CYCLE AND MOTILITY p53, p21, p38 G2/M ERK1/2, STAT3 Cyclin D1 Wnt/β-catenin, EMT markers MMP-2, u-PA,	Damnacanthal and Nordamnacanthal (*Morinda citrifolia* L.) Compound CID: 2948 Compound CID: 160712	Apoptosis; disrupts migration; inhibits cell proliferation in oral cancer cells; a promising experimental antitumor agent for oral squamous cell carcinoma cells therapy [259].
	Epigallocatechin-3-gallate (EGCG), (green tea) Compound CID: 65064	Inhibits proliferation and migration, promotes apoptosis; reduces tumor volume and size in vivo; potent candidate for oral cancer prevention and treatment [45].
	Fucoidan Compound CID: 204	Stops G2/M cell cycle, inhibits EMT markers, suppresses migration, reducing oral cancer cell proliferation and tumor growth in vivo; potential as a nutraceutical for oral cancer prevention and treatment [6].
	Gallic acid (GA) Compound CID: 370	Significant apoptosis in oral cancer cells by inducing DNA damage and impairing DNA repair mechanisms [260].
	Genistein, cinnamaldehyde, resveratrol	It inhibits pro-inflammatory cytokines LPS, TNFα, IL1, IL6, COX-2, LOX, reduces oxidative stress, Bcl-2, Bax and lowers the metastasis of cells. It also downregulates KIF20A, ERK1/2, cIAP-2, survivin, cyclin D1, suppresses matrix metalloproteinase (MMP)-3, MMP-9, COX-2, VEGF and STAT3 [131].
	Goniothalamine (GTN) (*Goniothalamus macrophyllus* Hook. f. & Thomson) Compound CID: 6440856	Inhibits proliferation of oral cancer cells through apoptosis, involving cytochrome c release, disruption of mitochondrial membrane potential and activation of caspases [261].
	Honokiol (*Magnolia officinalis* Rehder & Wilson) Compound CID: 72303	Decreases the Wnt signaling transducers such as *β*-catenin and TCF-4 increased GSK-3*α*/*β*, a *β*-catenin degradation promoting kinase. Consequently, the protein products of *β*-catenin downstream genes such as *c-Myc* and *Cyclin D1* were downregulated; CD44, was also inhibited; promising candidate for targeted oral cancer therapy [262].
	Pterostilbene Compound CID: 5281727	Reduces migration, invasion and metastasis; the activities and protein levels of the MMP-2 and urokinase-type plasminogen activator (u-PA) was inhibited; chemopreventive agent against oral cancer metastases [239].
	Luteolin Compound CID: 5280445	Inhibit crucial proteins in OSCC by molecular docking; reducing p38 phosphorylation and downregulating matrix metalloproteinase (MMP)-2 expression; promising candidates for the development of targeted anti-cancer therapies against OSCC [238].
	Hinokitiol Compound CID: 3611	Induces cytotoxicity against cancer cells with low toxicity to normal cells against OSCC [263].

Although most phytochemical compounds exhibit minimal toxicity, their clinical application may be limited because of their bioavailability due to poor solubility. The bioactive potential could be affected during the release, absorption, distribution, metabolism and excretion steps after their oral ingestion [218,264].

**Table 5:** Research stages of synergistic effects of phytochemicals in combination with chemopreventive agents.

Phytochemicals + Chemopreventive Agents	In Vitro Cell Culture	In Vivo Studies
Curcumin analog (PAC) + Cisplatin [265,266,267]	Concentrations used: cisplatin (from 0.1 M to 1 M), PAC (2.5 and 5 M). Ca9-22 oral cancer cells’ apoptosis; enhanced autophagy, ROS; reduced mitochondrial membrane potential.	Inhibited tumor angiogenesis in head and neck cancer cells [268].
Caffeic acid phenylethyl ester (CAPE) + 5-FU [269,270]	TW2.6 cells with CAPE and 5-fluorouracil (5-FU) lead to additive inhibition of cell proliferation. CAPE treatment does not affect the proliferation of normal human oral fibroblast (NHOF) cells at a concentration lower than 100 μM, suggesting that CAPE exhibits a selective suppressive effect on human oral cancer cells.	CAPE treatments have been shown to sensitize cancer cells to chemotherapeutic drugs and radiation treatment by inhibiting pathways that lead to treatment resistance in animal models.
Thymoquinone (TQ) + Cisplatin (CDDP) [271]	Cell-killing effect of TQ, CDDP and their combination against UMSCC-14C and OEC cell lines was observed with a concentration range of 0.01–100 μM. Additive or synergistic effects, significantly increasing cancer cell apoptosis, with a near-total apoptosis rate of 99.3%.	TQ ameliorates the drugs complications, as it improves the CDDP-induced nephrotoxicity in animal models, most probably by its antioxidant activity.
Apigenin + oxaliplatin (OXA) [272]	The combination of apigenin (40 µM) and a low concentration of OXA (5 µM) on OSCC revealed that apigenin could inhibit the pro-tumor metastatic effect of OXA through downregulating the expression of LINC00857.	Not yet tested in animal models.
Anethole + Cisplatin [273]	Anethole (3 µM) potentiated cisplatin-induced inhibition of Ca9-22 gingival cancer cells, reduced cell viability, increased apoptosis and inhibited key cancer signaling pathways including MAPK, β-catenin and NF-κB.	Not yet tested in animal models

In contrast, combinations of bioactive compounds and chemopreventive agents used for successful therapeutic applications provide an important source of pharmaceutical compounds (Table 5) with novel mechanisms of action useful in current drug development, some of which are in various clinical trials [265,273,274].

In addition, combination therapy offers a promising alternative for better disease management, e.g., cisplatin and curcumin analogue (PAC) have been shown to be effective in attenuating resistance to cisplatin therapy. The data presented indicate that PAC has the potential to serve as a powerful complementary agent to cisplatin in the treatment of gingival squamous cell carcinomas [265]. In an experimental study on 78 subjects (35–70 years old) with a treatment protocol based on curcuminoids 100 mg, three times a day for 60 days, from weeks 4–16 post-surgery, a decreasing nephrotoxicity incidence by 9% was found, together with an anti-inflammatory and antioxidant possible underlying mechanism of curcumin [266]. A study on human and rat glioblastoma cell lines showed that curcumin can overcome their radioresistance and chemoresistance. Also, in this case, curcumin was able to sensitize glioma cells to cisplatin by decreasing the expression of BCL-2 and members of the IAP family, as well as DNA repair enzymes. Another preclinical study reported that curcumin was able to inhibit the expression of NF-κB, which is a target for the induction of cell death in this cancer, in synergy with cisplatin [267].

These synergistic effects have been shown as a potential treatment in suppressing various mechanisms and proteins involved in cell invasion, proliferation and metastasis [267]. An in vivo growth inhibitory effect of liposomal curcumin-difluorinated (CDF) was evaluated in the nude mice xenograft tumor model of UM-SCC-1R and the inhibition of CD44 was measured. Treatment of the resistant cell lines in vitro with liposomal CDF resulted in a statistically significant growth inhibition (*p* < 0.05). The nude mice xenograft study showed a statistically significant tumor growth inhibition of UM-SCC-1R cells and a reduction in the expression of CD44 (*p* < 0.05), indicating an inhibitory effect of liposomal CDF on CSCs [268].

A study was carried out to investigate the effect of 6-shogaol, a bioactive compound of ginger, on the growth of OSCC cells by cell viability and soft agar colony formation assay. Migration and invasion assays were conducted to confirm the effect of 6-shogaol on OSCC cell metastasis. Apoptosis was detected by flow cytometry and the underlying mechanism on the antigrowth effect of 6-shogaol in OSCC cells was assessed using Western blotting. As a result, 6-shogaol not only suppressed proliferation and anchorage-independent cell growth in OSCC cells, but also induced apoptosis by regulating the apoptosis-associated factors such as p53, Bax, Bcl-2, and cleaved caspase-3. Migration and invasion of OSCC cells were inhibited following the regulation of E-cadherin and N-cadherin by 6-shogaol. Additionally, 6-shogaol treatment significantly inhibited the PI3K/AKT signaling pathway [229].

A study demonstrated that nimbolide, a neem limonoid, induced stereotypical changes in oral cancer cells characteristic of both apoptosis and autophagy. Time–course experiments revealed that nimbolide induces autophagy as an early event and then switches over to apoptosis. Nimbolide negatively regulates PI3K/Akt signaling with consequent increase in p-GSK-3βTyr216, the active form of GSK 3β that inhibits autophagy. Downregulation of HOTAIR, a competing endogenous RNA that sponges miR-126, may be a major contributor to the inactivation of PI3K/Akt/GSK3 signaling by nimbolide. Analysis of key markers of apoptosis and autophagy as well as p-AktSer473 during sequential progression of hamster and human OSCC revealed a gradual evolution to a pro-autophagic and antiapoptotic phenotype that could confer a survival advantage to tumors [248]. Using different natural extracts and compatible biocompounds (chitosan) mixtures with various application methods (sponges, patches, films) creates the opportunity to administer mucoadhesive products with promising effects on anticancer treatment [275,276,277]. When those combinations are administered, the dose and side effects of these chemotherapeutic drugs are reduced. Unfortunately, combination therapy is still a challenge in medical science today, leading researchers to find the best therapeutic response with the fewest side effects [269,278,279].

Current therapeutic approaches presented in some in vitro and in vivo studies also included combinations of plant compounds and conventional agents known as anticancer drugs (Table 5). This suggests that there are sufficient scientific and procedural arguments, which denotes that clinical stages can be approached because of the numerous benefits for the patient, including reducing the adverse effects induced by cytostatic drugs. Therefore, some limitations of those studies may include the fact that the concentrations required for anticancer activity in vitro may not always be feasible, practical, or effective in clinical settings.

### 4.2. Toxicity Considerations in Phytochemical-Based Cancer Treatments

Evidence from laboratory and epidemiologic studies suggests that phytochemicals may reduce cancer risk. On the other hand, however, certain phytochemicals may act as carcinogens, neurotoxic, nephrotoxic, hepatotoxic or tumor-promoting agents [280,281].

Phytochemical toxicity comes from the triggering of various mechanisms. Oxidative stress, involved in the damage of cellular components can be induced because of reactive oxygen species (ROS) generated during metabolism [280]. Some compounds may interfere with enzymes, thus realizing disruption of essential biochemical pathways. Understanding these mechanisms is essential for risk assessment and risk management [282]. In cases of excessive consumption, accumulation of toxic metabolites may occur, which may lead to adverse effects. Toxicity may also be potentiated by possible interactions between phytochemicals (flavonoids, terpenoids), drugs [282].

Researchers and health practitioners, by elucidating the balance between beneficial and potentially toxic effects, can guide safe consumption and capitalize on the positive aspects of phytochemicals while minimizing risks [282].

## 5. Conclusions

Carcinogenesis is a multi-step process closely linked to molecular changes induced by multiple risk factors. Oral cancer represents a main public health issue due to the complexity, the lack of effective and potent drugs, the multitude of side effects and the expensive treatments and mortality rates. The phytochemicals studied contributed to the understanding of the processes influencing the anti-invasive and antimigratory capabilities of OSCC, with an inhibitory effect on metastatic oral cancer cells by limiting genes’ expression and activity through a decline in the signaling pathway. In addition, it contributed to an improved understanding of the ability of phytochemicals to exert additive and synergistic effects, providing new insights into their potential efficacy in the management of OC. Also, the side effects of commonly used chemotherapeutic treatments may be ameliorated, providing a better quality of life for cancer patients. The above discussion strongly suggests that this herbal compound should be further explored as a potential complementary oral cancer treatment option for clinical trials in both the prevention and treatment of oral cancer. Further research should be directed towards prioritizing clinical trials that could be aimed at achieving therapeutic efficacy and safety in terms of the associated administration of the bioactive compounds found in this review. This combination could lead to a wider scope of medicines, by integrating these natural compounds into optimized treatments to improve patient outcomes.

## Figures and Tables

**Table 3:** Summary of extracts and their mechanisms in oral cancer effects.

Family and Scientific Name of the Plant		Molecular Targets	Molecular Mechanism
Apiaceae	*Trachyspermum ammi* L. [179]	ROS, SOD, CAT, GPx and GSH), EtBr/AO and TBARS staining	It exhibits significant cytotoxicity and antioxidant activity; anticarcinogenic activity by promoting ROS generation by modulating the induction of apoptosis in oral cancer KB KB cell.
	*Centella asiatica* L. [180]	ROS	Increases intracellular ROS generation, indicating that ECa 233 activates cells death by apoptosis associated with ROS generation; induces cell cycle under G1 in KON cells.
	*Cynara scolymus* L. [181]	Bax, Caspase-9, Bcl-2	G2/M phase cell cycle arrest, increase in pro-apoptotic genes, decrease in anti-apoptotic genes; no negative effects on normal cells; a potentially safe and effective anticancer agent for oral cancers.
Anacardiaceae	*Tapirira guianensis* Aubl. [182]	MMP-2, FAS, Caspase-3, cleaved PARP	Inhibits migration, matrix degradation and invasion; activates extrinsic apoptosis pathway; potential as anticarcinogen.
Clavulariidae	*Clavularia inflata* [183]	ROS, Caspase 3/8/9	Preferential antiproliferation against oral cancer cells; effects of increasing Annexin V, activating Caspase, increasing ROS, increasing MitoSOX, decreasing GSH, increasing DNADamage.
Clusiaceae	*Garcinia mangostana* L. [184]	Caspase 3 and 9, Bax, Bcl-2	Apoptosis in oral cancer cells; upregulated the expression of pro-apoptotic proteins, including caspases and Bax, and downregulated the expression of anti-apoptotic protein Bcl-2; therapeutic potential, anticancer agent against OSCC.
Commelinaceae	*Commelina* sp. [185]	Caspase 3, 8, 9, Annexin V, γH2AX, 8-OH-dG	Prefer antiproliferation and apoptosis have been associated with cellular and mitochondrial oxidative stress in oral cancer cells.
Cupressaceae	*Juniperus communis* L. [186]	p53/p21, Rb signaling	Promotes apoptosis via intrinsic and extrinsic pathways; enhances reduction in cancer cell viability when combined with 5-fluorouracil; used as adjuvant therapy.
	*Juniperus squamata* Buch-Ham [187]	Mcl-1, Bcl-xL and Bcl-2	It has cytotoxic effects; the mitotic catastrophe may facilitate G2/M arrest as well as apoptotic cell death in human oral cancer cells; potential therapeutic agent.
Ericaceae	*Rhaponticum uniflorum* Hutch. & F.K. Ward [188]	E-cadherin, Prx1, Vimentin, Snail	Inhibits proliferation, induces apoptosis; suppresses migration and invasion by suppressing Prx1 expression and the EMT process in OSCC; reduces tumor growth in OSCC.
Gracilariaceae	*Gracilaria tenuistipitata* var. liui [189]	Caspase, MMP, ROS	Anti-proliferative effect against oral cancer cells by inducing apoptosis and modulating oxidative stress (increased ROS), decreased glutathione (GSH); mitochondrial membrane potential (MMP) was significantly reduced; natural product extract promising for potential use in oral cancer therapy
Lamiaceae	*Rosmarinus officinalis* L. [190]	ROS, G2/M phase, autophagosome formation	Stopping the cell cycle, promoting apoptosis and inducing autophagy; Apoptotic changes as cell cycle arrest at G2/M phase; enhances ROS expression in cancer cells; a promising approach for OC management; potentially reducing side effects of conventional chemotherapy.
Lauraceae	*Persea americana* Mill. [191]	EGFR, c-RAF, ERK1/2	Inhibits phosphorylation in the EGFR/RAS/RAS/RAF/MEK/ERK1/2 cancer pathway, reduces oral cancer cell proliferation.
	*Cinnamomum cassia* [192]	Caspase-3, Bcl-2	Apoptosis (Caspase-3 activation, Bcl-2 reduction, and phosphatidylserine inversion) and autophagy; decreases tumor volume, weight and Ki-67 expression in vivo, indicating potential in oral cancer treatment.
	*Cryptocarya concinna* Hance [193]	ROS, mitochondrial dysfunction, Annexin V	Antiproliferative potential against oral cancer cells involving apoptosis, ROS generation and membrane depolarization of mitochondria.
Magnoliaceae	*Magnolia* sp. [194]	AMPK, STAT3, ROS	Demonstrates chemopreventive efficacy by inhibiting mitochondrial respiration; increases ROS generation leading to Prx oxidation; inhibits tumor growth; inhibits 4NQO-induced oral carcinogenesis; exhibits chemopreventive effects in OC.
Moringaceae	*Moringa oleifera* Lam. [195]	HSF1	Anti-proliferation and cell cytotoxicity; inhibits the phosphorylation of protein kinase C (PKC), which regulates protein transcription (HSF1); effective as a safe anti-cancer agent.
Myristicaceae	*Myristica fragrans* Houtt. [196]	Intrinsic pathway markers	Apoptosis via the intrinsic pathway; Bcl-2 protein expression reduced after treatment; a promising candidate for oral cancer chemoprevention.
Nepenthaceae	*Nepenthes* hybrid [167,197]	ROS, γH2AX, 8-oxo-2′-dezoxiguanosine	Apoptosis; antiproliferative effects; potent anticarcinogenic properties against oral cancer cells; intracellular ROS and mitochondrial superoxide (MitoSOX) were overexpressed and mito chondrial membrane potential (MMP) was disrupted; DNA damages such as γH2AX and 8-oxodG were increased; promising candidate for oral cancer therapy.
Nyctaginaceae	*Boerhaavia diffusa* L. [198]	ADB, Bcl-2, Caspase	Shows a promising cytotoxic effect in oral cancer cells that induces apoptosis by regulating antiapoptotic proteins and by blocking DNA synthesis; inhibits cell proliferation in a dose-dependent manner.
Plumbaginaceae	*Plumbago zeylanica* L. [199]	NF-κB, Cyclin D1, p53	Induces apoptosis in OSCC cell lines; inhibits the growth of oral cancer cell lines; stops the cell in the G2/M phase of the cell cycle; a strong candidate for chemoprevention and OSCC chemotherapy.
Portulacaceae	*Portulaca oleracea* L. [200]	Bax, Bcl-2	Induced apoptosis, reduced cell migration and invasion; exhibited significant cytotoxic effects. Potential anti-proliferative effects on oral cancer cells; could serve as an additional therapy alongside conventional chemotherapeutic agents;
Punicaceae	*Punica granatum* L. [201]	MMP-2, MMP-9, ATP, antioxidant gene	A potential therapeutic agent for oral cancer. Anti-proliferative, anti-angiogenesis and pro-apoptotic processes; reduces ATP formation, shortens the subG1 phase and increases apoptosis; may be used as a candidate for oral cancer therapy.
Rosaceae	*Duchesnea indica* Andr. Focke [202]	MMP-2, MEK/ERK	Inhibits motility, migration and invasion of OSCC cells by decreasing MMP-2 protein expression and activity through suppression of the MEK/ERK signaling pathway, effectively reducing their metastatic potential.
Salvadoraceae	*Salvadora persica* L. [203]	Apoptotic markers	Cytotoxicity and apoptosis in oral cancer cells with minimal effects on normal cells; cancer preventive, effects in the oral cavity
Solanaceae	*Lycium barbarum* L. [204]	ERK1/2, AKT1, Cyclin D1, CDH2, VIM, CDH1	Inhibits cell proliferation, migration and epithelial–mesenchymal transition (EMT); reduces the phosphorylation of ERK1/2 and AKT1 with concomitant downregulation of CCND1, CDH2, and VIM and upregulation of CDH1 expression anticarcinogenic properties against OSCC.
	*Withania somnifera* L. Dunal [205]	BRD3, CDK2, Withanolides	Apoptosis, accumulates cells in subG1 phase; cytotoxicity comparable to standard drugs; promising source of therapeutic agents for oral cancers
Theaceae	*Camellia sinensis* L. [206,207]	EGFR, VEGF, Cyclin D1	Effective chemopreventive agent for oral cancer with the potential to reduce the risk of malignant transformation of oral lesions.
Usneaceae	*Usnea barbata* (L.) Webber ex F.H. Wigg. [207]	ROS, MitoSOX, MMP	Preferred causes kills oral cancer cells and is associated with oxidative stress, apoptosis and DNA damage.
Tricholomataceae	*Tricholoma matsutake* [208]	Bak protein	Inhibits growth and induced apoptosis, as demonstrated by poly (ADP-ribose) polymerase (PARP); increased Bak protein expression, while Bax, Bcl-XL and Mcl-1 were not affected; nuclear cleavage and condensation and fragmentation;
Theonellidae	*Theonella swinhoei* [209]	Caspase 3/7/8/9, γH2AX, 8-OH-dG	Apoptosis; Inhibits cell proliferation of oral cancer cells but does not affect normal cells viability; preferential anti-proliferation function.

## Data Availability

Data are contained within this article.
